# Topical Oxygenation Improves Microvascularity in a Human Ex Vivo Palatal Tissue Model: A Histological Analysis

**DOI:** 10.3390/dj13020077

**Published:** 2025-02-10

**Authors:** Andrea Pilloni, Cira Rosaria Tiziana Di Gioia, Raffaella Carletti, Gerarda D’Elia, Michaela Di Mario, Angela Molendini, Umberto Romeo, Lorenzo Marini

**Affiliations:** 1 Department of Oral and Maxillofacial Sciences, Sapienza University of Rome, Via Caserta 6, 00161 Rome, Italy; andrea.pilloni@uniroma1.it (A.P.); umberto.romeo@uniroma1.it (U.R.); 2 Department of Radiological, Oncological and Pathological Science, Sapienza University of Rome, 00161 Rome, Italy; cira.digioia@uniroma1.it (C.R.T.D.G.); raffaella.carletti@uniroma1.it (R.C.)

**Keywords:** wound healing, topical oxygen therapy, histological analysis, immunohistochemical analysis, angiogenesis

## Abstract

**Background:** Several therapies involving the use of oxygen have been developed; however, the literature to date has poorly addressed the effects of Topical Oxygen Therapy (TOT) on oral tissues. The aim of the present pilot study was to histologically evaluate the response to TOT in epithelial–connective samples harvested from the palate. **Methods:** In eight patients selected for a free gingival graft (FGG) procedure, the lateral portions of the graft were randomly assigned to receive TOT (test) or no treatment (control). Both the portions were stained with hematoxylin–eosin for the evaluation of histomorphological changes and with Picrosirius for the study of collagen. Immunohistochemical staining was performed with antibodies for the characterization of the inflammatory infiltrate and neoangiogenesis. **Results:** The analysis failed to show morphological variations in both groups, demonstrating that there was no tissue damage due to TOT. The prevalence of type I collagen in both samples supported this finding. Immune cells were present in low levels and mainly consisted of CD3+ T lymphocytes. The histomorphometric analysis showed an increased average vessel area (7607.95 μm^2^ ± 3983.24 vs. 4038.42 μm^2^ ± 1823.52), an increased number and caliber of vessels (49.82 ± 20.55 vs. 32.35 ± 16.64), and a higher microvessel density (7.89 ± 3.25 vessels/0.26 mm^2^ vs. 5.13 ± 2.63 vessels/0.26 mm^2^) in the test group. **Conclusions:** Although further investigations are needed, TOT could improve and speed up oral wound healing through the local condition of hyperoxia.

## 1. Introduction

Wound healing is a dynamic process involving numerous cell types and metabolic mediators, which must act according to a well-organized chronology of biological events that are crucial for the quality of the final repair of injured tissue [1].

Oxygen plays a crucial role in the wound healing process [2]. Tissue repair demands increased energy, which is closely associated with the availability and function of oxygen at the wound site. A reduction or absence of oxygen can lead to a slowdown or complete halt of healing. Interestingly, temporary hypoxia following a wound acts as a signal that activates various healing mechanisms, including the production of cytokines and growth factors by macrophages, keratinocytes, and fibroblasts, such as platelet-derived growth factor (PDGF), transforming growth factor beta (TGF-β), vascular endothelial growth factor (VEGF), tumor necrosis factor alpha (TNF-α), and endothelin-1 [3]. On the other hand, prolonged or chronic hypoxia is not able to sustain angiogenesis in wounds because cells are deprived of a crucial source of energy, thus delaying the healing and leading to tissue loss [4]. Regrettably, wounds often occur in tissues where oxygen levels may already be diminished. Conditions such as diabetic foot ulcers, pressure sores, venous stasis, and arterial ulcers are all associated with impaired hemodynamics and reduced oxygen delivery to tissues [5,6].

Since the 1960s, several therapies involving the use of oxygen have been developed for the treatment of wounds and for improving their healing; many studies have demonstrated the positive effects of therapies such as Hyperbaric Oxygen Therapy (HBOT), Topical Oxygen Therapy (TOT), and Continuous Oxygen Diffusion (CDOT). Currently, HBOT is a treatment approved exclusively for chronic wounds and requires careful preliminary examination and monitoring of patients. TOT delivers oxygen directly to the tissue to be treated, limitedly improving the hypoxic condition at the wound bed. The other advantages of TOT are that it uses simpler instruments, is cheaper than HBOT, and does not involve any risk of toxicity or complications related to hyperbaric oxygen [7,8,9].

TOT has demonstrated significant benefits in wound healing across various types of wounds, including diabetic foot ulcers (DFUs), pressure ulcers, and venous ulcers [10]. Studies have consistently shown that TOT as an adjuvant treatment leads to higher rates of wound healing, likely due to enhanced tissue oxygenation, which supports critical processes like angiogenesis, bacterial elimination, and collagen synthesis [11]. Oxygen plays a key role in these processes by promoting the inflammatory phase of healing and fostering the formation of new blood vessels. Although the time to complete healing showed mixed results across studies [12,13], a reduction in wound area was universally observed in trials using TOT [14]. To date, the effects of TOT on oral tissues have been poorly investigated.

Recently, an ex vivo model using fresh human skin explants has been adopted across various medical fields [15,16]. This model, which does not rely on traditional culturing techniques, offers substantial potential for investigating different skin pathologies. Its key advantage lies in its ability to preserve physiological local responses. However, it is important to note that this ex vivo approach is limited by the absence of blood circulation, meaning the inflammatory and vascular responses observed are confined to the local reactions of skin-resident cells, without accounting for systemic effects.

To our knowledge, this study is the first to apply an human ex vivo model to oral epithelial and connective tissues harvested from the palate specifically to histologically assess the response of palatal tissue to TOT.

## 2. Materials and Methods

### 2.1. Study Design

**Figure 1:**
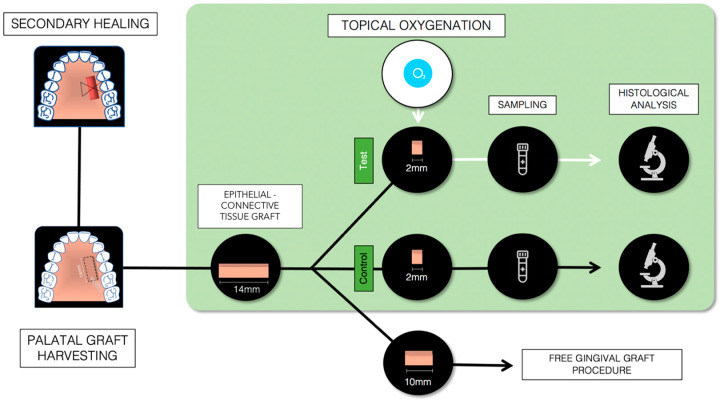
Flowchart of the study.

The present study is a pilot histological study using a human ex vivo palatal tissue model. The epithelial–connective tissue harvested from the palate for a free gingival graft (FGG) procedure was divided into three parts. The two lateral parts, not necessary for the keratinized tissue augmentation procedure, were randomly assigned to the test group (treatment with TOT) or to the control group (without oxygen) and subjected to histological analysis (Figure 1).

Patients were enrolled and treated at the Section of Periodontics of the Department of Oral and Maxillofacial Sciences of Sapienza University of Rome from March 2022 to July 2022. Histological analysis was conducted at the Department of Radiological, Oncological and Pathological Science of Sapienza University of Rome.

The study protocol was approved by the Ethics Committee of the Sapienza University of Rome (protocol code 0155/2022; date of approval: 3 March 2022). Clinical procedures were performed in accordance with international Good Clinical Practice Guidelines and the Helsinki Declaration. Each patient provided written informed consent before being enrolled in the study and before each performed therapy.

### 2.2. Study Population

The study population consisted of patients recruited at the Periodontology Unit of the Department of Oral and Maxillofacial Sciences of Sapienza University of Rome. Patient selection was performed based on all of the following inclusion criteria:

- Age ≥ 18 years;
- Attached gingiva < 1 mm on at least one buccal surface of the mandibular incisors associated with/without poor oral hygiene and/or high frenulum insertion and/or gingival recessions;
- Full-mouth plaque score (FMPS) and full-mouth bleeding score (FMBS) < 15%;
- Periodontal health on intact or reduced periodontium.

Patients were excluded from the study in the presence of one or more of the following exclusion criteria:

- Smoking habits;
- History of neoplasia, radiotherapy, or chemotherapy for neoplasia;
- Pregnancy or breastfeeding in the previous 5 months;
- Intake of drugs or other therapies that affect the healing mechanisms (steroids, high-dose anti-inflammatory drugs);
- Diseases that influence tissue metabolism;
- Untreated periodontitis.

### 2.3. Tissue Samples and Human Ex Vivo Palatal Tissue Model

A standard-sized epithealial–connective tissue graft (14 × 7 mm) was harvested from the palatal area between the first premolar and the first molar at a distance of 2 mm from the gingival margin. The donor area was cleaned with sterile saline solution and sutured with 4-0 silk to stabilize the clot. The epithelial–connective tissue graft was prepared by removing any irregularities and adipose tissue and, if necessary, thinned to obtain a uniform thickness of approximately 1.5 mm.

The sample was divided into three parts. The central portion (10 × 7 mm) was used for a FGG procedure. It was carefully adapted and securely sutured to the recipient site, which was prepared to match the same dimensions as the central portion of the graft, using 6-0 monofilament nylon.

**Figure 2:**
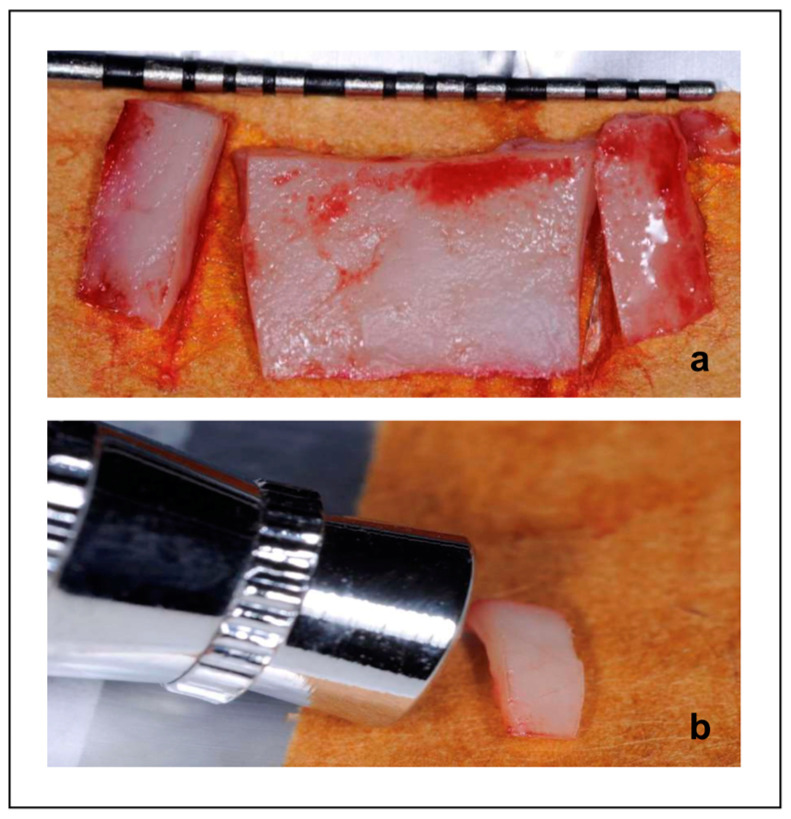
Extra-oral topical oxygenation. (**a**) The graft divided into three parts. (**b**) Topical oxygenation treatment.

The lateral portions of the graft (2 × 7 mm each) were randomized by tossing a coin to receive treatment with TOT (test) or no treatment (control) (Figure 2). Subsequently, both the portions were subjected to histological analysis.

### 2.4. Topical Oxygenation Treatment

TOT was administered using an oxygen generator (VIGO-PSA-6000, CaressFlow Srl, Bologna, Italy) that allows the delivery of pure O_2_ at 93 ± 3% for a flow rate of 6 L/min at atmospheric pressure (0.101325 MPa). The flow of O_2_ generated had a pressure of 0.04–0.07 MPa without reaching the parameters of a hyperbaric system (100% O_2_ in a closed system). Oxygen was delivered for 4 min through a special handpiece positioned at 2 mm from the tissue.

### 2.5. Histological Analysis

Within one hour of treatment, the surgical samples were fixed in 10% buffered formalin and embedded in paraffin. Consecutive formalin-fixed and paraffin-embedded histological sections (thickness: 3 μm) were stained with both hematoxylin–eosin (H&E) for the evaluation of morphological changes and Picrosirius red staining to specifically highlight collagen fibers in tissue samples. All the histological slides were analyzed using a light microscope. From the same paraffin blocks, further histological sections were performed for the immunohistochemical characterization of the inflammatory infiltrate and neo-angiogenesis.

For each specimen, the Picrosirius red staining was useful for detecting connective tissue damage because collagen is a major structural component of connective tissues like the extracellular matrix. Type I collagen, a predominant structural component of the extracellular matrix, is crucial for tissue integrity and repair. When tissue suffers damage, the prevalence of type I collagen can indicate the extent of the injury and the ongoing healing processes. Sections were analyzed using a light microscope under polarized light to evaluate the different types of collagen. Using a polarized light microscope, type I collagen appears yellow/orange, while type III collagen appears green.

The immunohistochemical analysis was performed with the following Leica ready-to-use antibodies:

- Anti-CD3 (T-lymphocyte-specific marker);
- Anti-CD20 (B-lymphocyte-specific marker);
- Anti-CD68 (macrophage-specific marker);
- Anti-CD34 (endothelial cell marker).

The reaction was amplified and visualized using the Leica Bond III immunostainer with Bond Polymer Refine Detection and 3,3′-diaminobenzidine (DAB).

Two independent pathologists (C.D.G. and R.C.) blinded to the treatment evaluated the intensity of the inflammatory infiltrate in 5 fields at 20× with the following scores: absent (0 positive cells), mild (1–10 positive cells), moderate (10–50 positive cells), and intense (>50 positive cells). The number of positive cells was expressed as mean ± SD.

The histological sections immunostained with anti-CD34 antibody were used to evaluate the following factors:

1. The number of vessels present in the subepithelial connective tissue (expressed as mean ± SD);
2. The caliber of the vessels (area occupied by the vessel in μm^2^, expressed as mean ± SD);
3. The microvessel density (expressed as the number of vessels on the total evaluated area) [17].

The immunostained slides were digitally acquired with an Aperio scanner (Leica Biosystems, Nussloch, Germany) and subjected to analysis using ImageJ software (version number 1.54k), quantifying the positive signal in 5 fields at 20× magnification (total area 6.31 mm^2^).

### 2.6. Statistical Analysis

As a pilot investigation, the primary objective was to assess feasibility and gather initial data on the potential biological and histological outcomes of TOT treatment of periodontal tissues. Consequently, a formal sample size calculation was not performed. Data are shown as means ± SEM (standard error of the mean). A two-tailed unpaired Student’s t test was used for statistical analysis. *p* value < 0.05 was considered statistically significant.

## 3. Results

Eight patients participated in the study, including three males and five females. The mean age was 38.3 ± 8.6 years, with an age range of 29 to 51 years. Individual demographic and histological data are presented in Table 1.

### 3.1. Histomorphological Analysis

**Figure 3:**
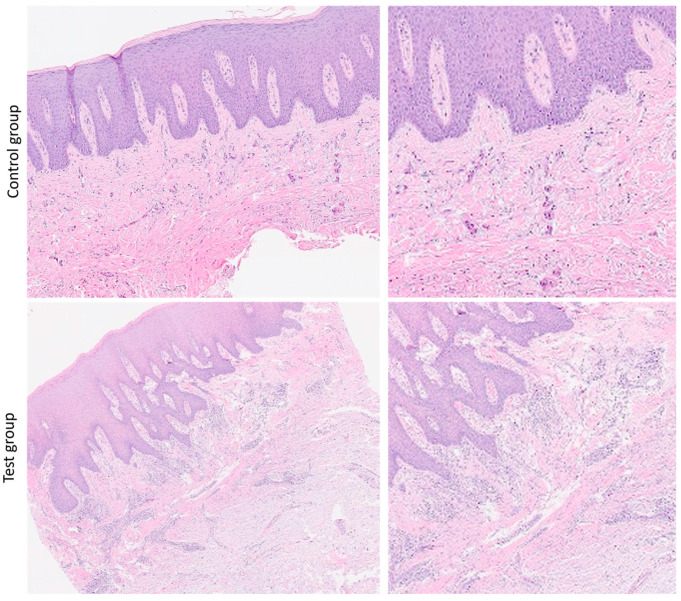
Representative microscopical sections of oral mucosa of control group and test group. Hematoxylin–eosin stains for each group: 10× (on left side) and 40× (on right side) magnification.

The microscopic analysis of the H&E-stained slides showed no significant differences in both the control group and the test group (Figure 3). In both groups, the oral mucosa showed a stratified squamous epithelium with mild hyperkeratosis (ortho- and parakeratosis), focal hypergranulosis, mild elongation of epithelial rete pegs and acanthosis; in all the specimens, there was no epithelial dysplasia. The subepithelial connective tissue was focally loose.

### 3.2. Polarized Light Microscopy

**Figure 4:**
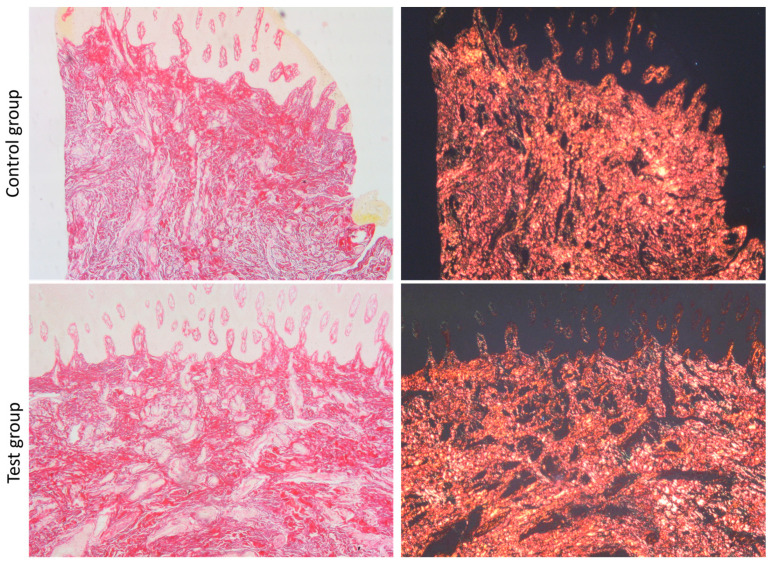
Microscopic view of Picrosirius-stained sections under a light optic microscope and a polarized light optic microscope (dark field) in the control group and the test group (20× magnification).

Sirius red-stained slides observed under the polarized light microscope showed the prevalence of type I collagen (orange/yellow in dark field) in the specimens of both the control group and test group (Figure 4).

### 3.3. Immunohistochemical Analysis

**Figure 5:**
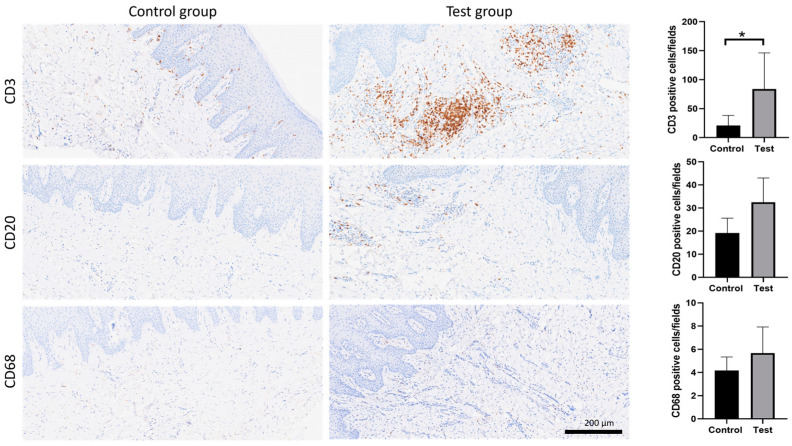
Immunohistochemical characterization of the inflammatory infiltrate: CD3+ T lymphocytes, CD20+ B lymphocytes, and CD68+ macrophages. (*: *p* < 0.05).

The immunohistochemical characterization of the inflammatory infiltrate showed a significant prevalence of CD3+ T lymphocytes, mostly in the test group compared to the control group (*p* < 0.05). There were some CD20+ B lymphocytes in the test group, while they were rarely observed in the control group. The CD68+ macrophage population was poorly represented in both groups (Figure 5).

**Figure 6:**
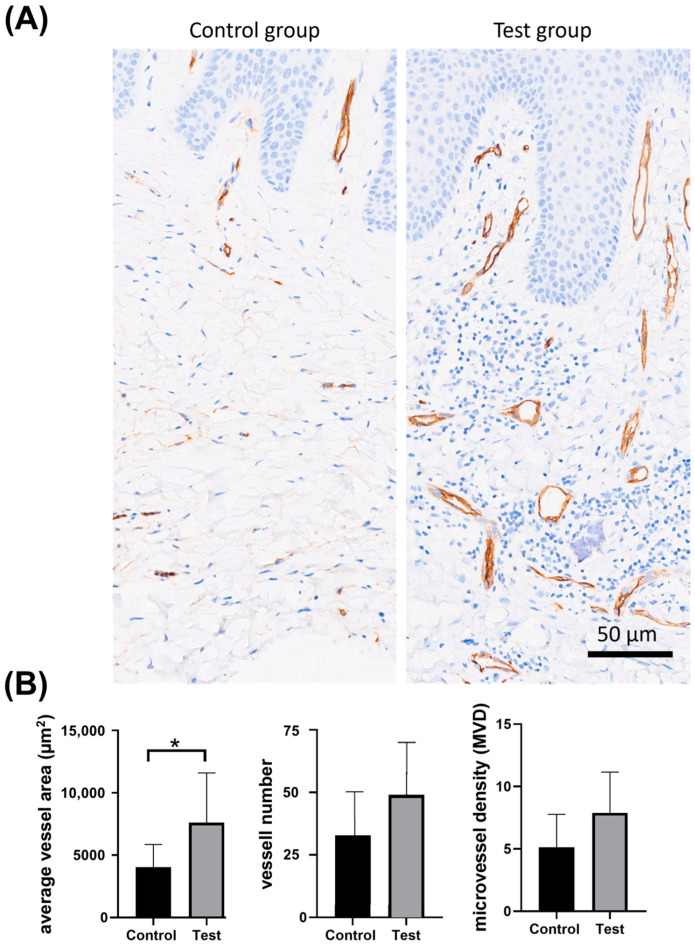
(**A**) Representative microscopic sections immunostained with CD34 antibody (brown stain) in the control group and test group. (**B**) Graphical representation of the vascular parameters in the control and test groups; *: *p* < 0.05.

Sections immunostained with anti-CD34 antibody showed a significantly increased average vessel area (7607.95 μm^2^ ± 3983.24 vs. 4038.42 μm^2^ ± 1823.52) in the test group vs. the control group. There was also an increase in the number of vessels (49.82 ± 20.55 vs. 32.35 ± 16.64) and a higher microvessel density (7.89 ± 3.25 vessels/0.26 mm^2^ vs. 5.13 ± 2.63 vessels/0.26 mm^2^) in the test group (Figure 6).

## 4. Discussion

The emphasis on techniques that enhance tissue oxygenation stems from oxygen’s critical roles in the wound healing process. In fact, elevated oxygen levels accelerate the healing of both soft and hard tissues by enhancing energy production, promoting epithelization, stimulating collagen synthesis, encouraging angiogenesis, facilitating bone repair, and supporting bactericidal activities. Consistently, hypoxia hinders the development of new blood vessels and decreases collagen levels by significantly elevating the production of collagenase-1 in fibroblasts [18].

Specifically, oxygen directly impacts anaerobic microorganisms and indirectly enhances the function of leukocytes, while also activating and supporting the body’s local defense mechanisms [19]. Furthermore, oxygen is a limiting substrate that produces reactive oxygen species (ROS) which perform an antibacterial function [20]. The elimination of bacteria from the wound site is also a process that relies on a high partial pressure of O_2_, which stimulates the respiratory burst. This burst activates the NADPH oxidase on the neutrophil membrane, leading to the production of superoxide. The superoxide then combines with oxygen molecules to generate reactive oxygen species (ROS), which play a key role in the bactericidal action [21].

Additionally, regenerative processes are enhanced by increased tissue capillarity and capillary vessel proliferation. Oxygenation stimulates collagen synthesis, supports fibroblast proliferation, and improves bone metabolism and turnover [22]. Hyperoxia can induce a strong angiogenic response that is self-sustaining, supporting the wound through the subsequent stages of the healing cascade [23]. Fries and co-workers evaluated the efficacy of TOT using a pre-clinical model of an excisional dermal wound in pigs. The results showed an increase in the partial pressure of oxygen for the test group, which was four times higher than the control group after 4 minutes of application, and the wound closure was accelerated after multiple treatments. In addition, histological data revealed that the wounds benefited from the treatment, showing a greater presence of VEGF, better angiogenesis, and advanced tissue process with higher-quality collagen compared to the control group [24].

While there is existing evidence in the literature regarding the impact of TOT on extra-oral wound healing [12,13,14], oral tissues have been poorly investigated in this context. Schlagenhauf et al. assessed the effect of repeated subgingival oxygen irrigations in previously untreated deep periodontal pockets. The re-evaluation of all clinical and microbiological parameters at the end of the study revealed that repeated oxygen insufflations resulted in a significant clinical improvement of the periodontal baseline conditions superior to controls [25]. More recently, Gaggi et al. showed that adjunctive oxygen therapy results in the early eradication of pathogenic anaerobic microorganisms and reduced periodontal tissue destruction in cases of acute necrotizing periodontal disease [26].

Conversely, a higher number of studies on the effects of oxygen therapies in dentistry have been published, but they have primarily focused on HBOT. Gajendrareddy and co-workers initially induced periodontitis in rats and subsequently subjected them to HBOT for 2 h twice a day. At 28 days, the results showed a higher expression of type I collagen with a more consistent deposition, maturation, and robustness of these fibers. On the other hand, no difference in the inflammatory response was observed [27]. Other studies have evaluated the effects of HBOT in patients with periodontitis, demonstrating that the number of microorganisms present inside the periodontal pocket were reduced in the test groups and that HBOT combined with scaling and root planing was the most advantageous treatment for periodontitis [28,29,30]. Finally, Shennon and co-workers treated 40 guinea pigs after making wedge-shaped gingival excisions for 90 min per day under the conditions of different oxygen percentages and pressure values (20.8% O_2_ at 2.4 atm; 100% O_2_ at 1 atm; and 100% O_2_ at 2.4 atm). The group treated with O_2_ at 2.4 atm showed more advanced healing, evidenced by an early maturation of the wound, while the group that received O_2_ at normobaric conditions presented a long junctional epithelium [31].

This pilot study is the first to assess the histological effects of TOT in a human ex vivo palatal tissue model. The chosen investigation methods were based on the fact that epithelial and connective tissues retain vitality and responsiveness for a short time between sampling and fixation in formalin, even when isolated from the organism. The focus was on the cells most likely to be stimulated by the treatment, including endothelial and immune cells. Furthermore, potential morphological changes in the tissues, such as the number, caliber, and density of the vessels, were evaluated.

The results were noteworthy, starting with the histomorphological analysis of the epithelial–connective samples stained with hematoxylin–eosin, which showed no morphological variations in either the test or control groups. This finding indicates that the treatment with topical oxygen and the subsequent fixation in formalin did not result in any tissue damage. Furthermore, histochemical characterization of the inflammatory infiltrate revealed that immune cells were present, but in low levels and mainly consisting of CD3+ T lymphocytes. CD20+ B lymphocytes were rare and CD68+ macrophages were scarce. These cells remained confined to the canonical locations, id est below the epithelium and around the capillary vessels.

The more intriguing results pertained to the analysis of vessel number and caliber, showing an increased microvessel density in the test group compared to the control group. These results are consistent with those presented by Fries and co-workers [24]. Interestingly, in the present study, the test group exhibited the highest levels of CD3+ T lymphocytes, which coincided with the greatest number of microvessels and microvascular density. This suggests that the improved microvascularity may not only be a result of the direct effects of hyperoxia but also due to angiogenesis induced by CD3+ T lymphocytes through VEGF [32,33]. However, a plausible explanation for the observed increase in blood vessels may be the enlargement of existing vessels in response to localized oxygenation stimulation. This process could lead to the dilation of smaller, previously imperceptible vessels, making them visible under microscopic examination.

The findings of the present study are relevant from a clinical perspective. In fact, effective healing after mucogingival surgery depends on the rate of wound revascularization, along with the maintenance and restoration of the gingival tissue microvasculature [34]. Alterations in blood flow following soft tissue grafting may influence the wound healing process and the ultimate clinical results. Furthermore, analyzing blood volume at grafted sites could offer deeper understanding of how phenotype modification impacts peri-implant health, as inflammation is associated with changes in tissue perfusion [35]. Specifically, once an epithelial connective tissue graft is harvested, the healing of the recipient site relies on the re-establishment of collateral circulation from the periosteum and connective tissue bed. Blood flow begins from the superficial capillaries of the soft tissue grafts approximately 24 h post-surgery and continues for 4 to 5 days. Inadequate blood flow during this critical period can lead to ischemia, posing a serious risk. A new blood supply connection between the donor and recipient sites typically forms within 8 to 10 days [36]. In FGG procedures, both vertical and horizontal shrinkage of the graft can occur due to inadequate blood supply. This can negatively affect the expected outcomes, particularly in achieving sufficient keratinized tissue [37]. In this context, TOT could assist in preserving and regenerating the gingival tissue microvasculature, thereby reducing tissue contraction and enhancing the FGG procedure. Additionally, it could improve healing in various periodontal surgical techniques, particularly by promoting better vascularization, which is especially advantageous for secondary healing at the palate donor site.

However, this study has several limitations. Conducted ex vivo, the findings may not accurately reflect in vivo conditions due to differences in blood flow, immune responses, and tissue interactions. Additionally, a small sample size could limit generalizability. Nevertheless, given the exploratory nature of this study, it was deemed sufficient to provide preliminary insight into the potential effects of TOT on periodontal tissue healing, including its impact on microvascular density and tissue response. Another limitation is the lack of an air spray in place of the oxygen generator in the control group, which could have offered a more reliable comparison and further strengthened the methodological approach. Furthermore, the lack of long-term follow-up restricts understanding of the benefits of topical oxygenation beyond immediate healing. Finally, histological analysis provides only a snapshot of tissue characteristics, potentially missing dynamic healing processes.

## 5. Conclusions

Within the limits of this study, TOT seems not to determine any increase in the immune response and does not damage the exposed tissues. Enhancement of microvascularization in locally oxygenated tissues could promote optimal wound healing.

## Figures and Tables

**Table 1:** Demographic and histological data for each individual case.

Patient	Gender (M/F)	Age (Years)	Group	Number of Micro Vessels (in 5 Fields) (CD34)	Average Vessel Area (µm^2^) (CD34)	Microvessel Density (MVD) (in 1 Field) (CD34) 0.26 mm^2^	CD3- Positive Cells/Fields	CD20- Positive Cells/Fields	CD68- Positive Cells/Fields
1	F	51	Test	29	8.502	4.596	57	22	2
			Control	15.4	1.973	2.441	10	13	3
2	M	44	Test	65	7.591	10.301	30	34	4
			Control	15.4	1.973	2.441	13	25	5
3	F	36	Test	58	2.250	9.191	104	37	7
			Control	60	5.185	9.509	8	14	4
4	F	29	Test	79.6	15.564	12.615	201	38	7
			Control	47.8	2.679	7.575	20	13	3
5	F	36	Test	54	5.374	8.558	47	46	6
			Control	45	3.442	7.132	55	26	6
6	M	51	Test	22.6	5.404	3.582	64	18	8
			Control	29	7.064	4.596	19	24	4
7	F	31	Test	28.8	6.090	4.564	82	30	5
			Control	20	4.670	3.17	20	19	3
8	M	29	Test	61.6	10.080	9.762	83	34	6
			Control	26.2	5.321	4.152	21	19	4

## Data Availability

The raw data supporting the conclusions of this article will be made available by the authors on request.
